# Provider-initiated screening clinical breast examination and patient navigation in sub-Saharan Africa: protocol for an implementation mapping-based pilot study

**DOI:** 10.1186/s43058-026-01029-3

**Published:** 2026-07-25

**Authors:** Aragaw Tesfaw, Elima Jedy Agba, Edom Seife, Sara Debebe, Eyerusalem Getachew, Muluken Gizaw, Maureen Joffe, Adamu Addissie, Alex Mremi, Blandina Theophil Mmbaga, Eva Johanna Kantelhardt, Daniel S. O’Neil, Sefonias Getachew

**Affiliations:** 1 Network for Oncology Research in Africa (NORA), Global and Planetary Health Working Group, Institute of Medical Epidemiology, Biostatistics, and Informatics, Martin Luther University Halle-Wittenberg, Halle (Saale), Germany; 2 Department of Epidemiology & Biostatistics, School of Public Health, Addis Ababa University, Addis Ababa, Ethiopia; 3 Department of Public Health, College of Health Sciences, Debre Tabor University, Debre Tabor, Ethiopia; 4 International Research Center of Excellence, Institute of Human Virology, Abuja, Nigeria; 5 Department of Oncology, School of Medicine, College of Health Science, Addis Ababa University, Addis Ababa, Ethiopia; 6 South African Medical Research Council Aging African Adult Research Unit, University of Witwatersrand, Johannesburg, South Africa; 7 Department of Gynecology, Martin Luther University Halle-Wittenberg, Halle (Saale), Germany; 8 Kilimanjaro Clinical Research Institute, Moshi, Tanzania; 9 School of Medicine, KCMC University, Moshi, Tanzania; 10 Yale Cancer Center, Yale University, New Haven, CT USA

**Keywords:** Breast cancer screening, Patient navigation, Implementation strategies, Implementation research, Sub-Saharan Africa

## Abstract

**Background:**

Breast cancer care in sub-Saharan Africa is characterised by delays in time to presentation, diagnosis, and treatment initiation, due to low health-seeking behaviour, lack of access to care, long wait times, and weak referral systems with limited use of evidence-based interventions such as routine breast cancer screening and patient navigation (PN) services. To address these issues, we plan to develop, pilot, and evaluate a set of implementation strategies to integrate provider-initiated clinical breast examination (CBE) and PN into routine clinical practice to support timely breast cancer diagnosis and treatment initiation.

**Methods:**

We will conduct a pragmatic implementation pilot study in the four sub-Saharan African countries that make up the Network for Oncology Research in Africa (NORA) consortium: Ethiopia, South Africa, Nigeria, and Tanzania. The study will follow the implementation mapping (IM) process, drawing on theory, evidence, and stakeholders’ input to develop and evaluate a set of implementation strategies. Our target evidence-based intervention will be provider driven breast cancer screening via opportunistic, clinic-based CBE for all eligible patients and subsequent PN support for women with abnormal exams through diagnosis evaluation and initiation of treatment. All four countries will begin by conducting independent mixed-methods needs assessments structured according to the Consolidated Framework for Implementation Research to identify contextual barriers and implementation determinants. Working with local stakeholders, defined performance objectives and identified determinants will be used to select evidence-based implementation strategies. The set of strategies will then be implemented and iteratively evaluated in two phases, guided by the RE-AIM framework. Our primary implementation outcomes will address reach (the proportion of CBE uptake) and secondary implementation outcomes will address adoption by providers and implementation fidelity. Preliminary effectiveness outcomes will include stage distribution of newly diagnosed breast cancers and diagnostic and treatment intervals. Data sources will include existing medical records, internal study clinical records, structured facility resource surveys, patient and provider surveys, observational checklists, and qualitative interviews and focus group discussions.

**Discussion:**

This multi-country implementation study protocol demonstrates a structured, theory-informed approach to develop, pilot, and evaluate implementation strategies for breast cancer screening in diverse health care settings. If the intervention succeeds, findings from this pilot will inform design of future hybrid effectiveness–implementation studies, readiness for potential national scale-up, and adaptation to similar low and middle-income settings.

**Protocol registration number:**

The protocol is registered at clinicaltrials.gov: NCT07023198: registered 01 April, 2026, https://clinicaltrials.gov/study/NCT07023198.

**Supplementary Information:**

The online version contains supplementary material available at https://doi.org/10.1186/s43058-026-01029-3.

Text box 1. Contributions to the literature

• Unlike traditional top-down interventions, this implementation pilot study will use theoretical models and stakeholder input via the implementation mapping process to guide strategy development, increasing the likelihood of developing feasible and acceptable interventions.
• The study explores methods to select and tailor implementation strategies to address context-specific barriers and enhance facilitators, advancing knowledge on practical, evidence-informed approaches to implementation science.
• The study will be conducted in sub-Saharan African countries with diverse population and health systems from West, East and Southern Africa.
• Findings will contribute to the global implementation science literature by generating evidence on the feasibility of implementing provider-initiated clinical breast examination screening along with patient navigation across diverse health care settings in sub-Saharan Africa. Findings will inform national and global initiatives aimed at strengthening early breast cancer detection and care in low-resource settings and will provide transferable insights for adaptation and scale-up of similar interventions in comparable contexts.

## Introduction

Breast cancer remains a leading cause of cancer related mortality among women worldwide [1]. In 2022, there were 2.3 million new cases and 666,000 deaths globally, with 26.5% of cases and 12.2% of deaths occurring in sub-Saharan Africa (SSA) [2]. The breast cancer burden in SSA is projected to increase by 85.7% by 2040 [3]. Incidence and mortality rates vary significantly across SSA, with the highest age-standardized incidence rates reported in Nigeria (51.5 per 100,000 women per year) [4] while the rates of breast cancer in Ethiopia, South Africa, and Tanzania are estimated to be 40.8, 46.2, and 25.1 per 100,000 female population per year, respectively [2]. The five-year relative survival of breast cancer in SSA is less than 40% as compared to above 90% in high-income countries [5, 6]. These survival disparities are, in part, because delays in diagnosis and treatment remain common in SSA [7, 8].

The delays that patients with breast cancer in SSA experience in presentation and diagnosis have been attributed to low health-seeking behaviour, limited early detection practices along with limited health system provision of screening services, long waiting times for diagnostic workups, and weak referral systems [8–10]. As a result, most breast cancer is diagnosed at stage III or IV. For instance, 76% of patients in Nigeria [11] and 50–75% in South Africa presented with advanced-stage disease (stage III/IV) [12]. The World Health Organization (WHO) established the Global Breast Cancer Initiative (GBCI), with targets for improving breast cancer care in low- and middle-income countries (LMICs), which include strengthening early detection, with at least 60% of invasive breast cancers diagnosed at stage I-II, and timely diagnosis, with at least 80% of cancers diagnosed within 60 days of first presentation [13]. Unfortunately, the uptake of evidence-based interventions for earlier detection and diagnosis is limited in the region.

Although mammography is the standard tool for breast cancer screening, its use is limited in LMICs due to inadequate infrastructure, staff shortages, and high costs, leaving CBE as a potential alternative for screening. While the effectiveness of CBE as a standalone screening tool remains debated, studies suggest it can aid in early detection [14–16]. In a cluster-randomized trial from India, screening CBE every 2 years performed by trained providers decreased the proportion of stage III/IV cancers by 10% and, in a post-hoc analysis, also decreased breast-cancer related mortality in women greater than 50 years of age [17], supporting its role as a practical and accessible screening option for populations without access to routine mammography. Additional studies from Rwanda and Eswatini have shown improved early-stage detection by nurse-led CBE and the feasibility of integrating CBE into routine care [18, 19]. Provider-initiated CBE ensures that women who visit health facilities for any reason are opportunistically screened for breast abnormalities, increasing the chances of early detection. Meanwhile, patient navigation (PN) programmes for cancer patients, where health care providers (HCPs) guide patients through diagnostic and treatment pathways, increase screening rates, reduce delays in diagnosis, and improve patient adherence to treatment [20–22]. Integration of CBE and PN may meaningfully improve timeliness in detection, diagnosis, and initiation of care.

The Network for Oncology Research in Africa (NORA) is a multicounty collaborative consortium designed to strengthen cancer research, prevention, and care across sub Saharan Africa, bringing together partners in Nigeria, Ethiopia, South Africa, Tanzania, France, and Germany in collaboration with national ministries of health, the world health organization (WHO), universities, and African cancer centers and registries [23]. By linking these countries through a coordinated research and capacity building platform, NORA aims to generate high-quality evidence to guide national cancer control strategies and to reduce mortality by improving early detection, timely diagnosis, and treatment completion. One of the consortium’s main research tasks is to conduct a multi-country feasibility study to develop, pilot and evaluate a set of implementation strategies to integrate provider-initiated CBE and PN into existing health systems in Nigeria, Ethiopia, South Africa, and Tanzania. This protocol describes the planned development and piloting of an intervention that will be implemented based on each country’s national breast cancer guidelines, which all recommend the use of CBE in lower-level healthcare settings with referral for abnormal exams to higher-level diagnostic centres [24–27].

We will employ the Implementation Mapping (IM) process, a systematic, stepwise methodology that relies on an initial needs analysis and theoretical frameworks for the intentional selection of implementation strategies that are most likely to successfully support adoption and maintenance of an evidence-based intervention in a given context [28]. Guided by the IM process, each country will independently (1) design a suite of implementation strategies based on defined objectives and determinants for each implementation participant, (2) pilot and evaluate those strategies for feasibility, fidelity, adoption, and acceptability, and (3) generate preliminary evidence on its effect on diagnostic and treatment timeliness. In addition, the Consolidated Framework for Implementation Research (CFIR) framework will be used to guide the identification of multilevel implementation determinants and contextual factors that could affect the implementation process and outcomes by exploring five major domains: intervention characteristics, outer setting, inner setting, characteristics of individuals, and the implementation process [29]. The Behavior Change Wheel (BCW) framework which incorporates the capability, opportunity, motivation (COM-B) model at its core, will be used to systematic development of implementation strategies and to better understand behavioral determinants influencing the implementation practice within the local contexts [30].

By conducting this pilot through a multicounty consortium with diverse health systems and sociocultural settings, we will identify common and country-specific challenges that strengthen the generalizability of our findings. We hope to generate practical insights and scalable approaches to early breast cancer detection and diagnosis that can inform policymakers and be expanded nationally and regionally through integration with existing healthcare systems.

## Methods

### Study design

**Fig. 1:**
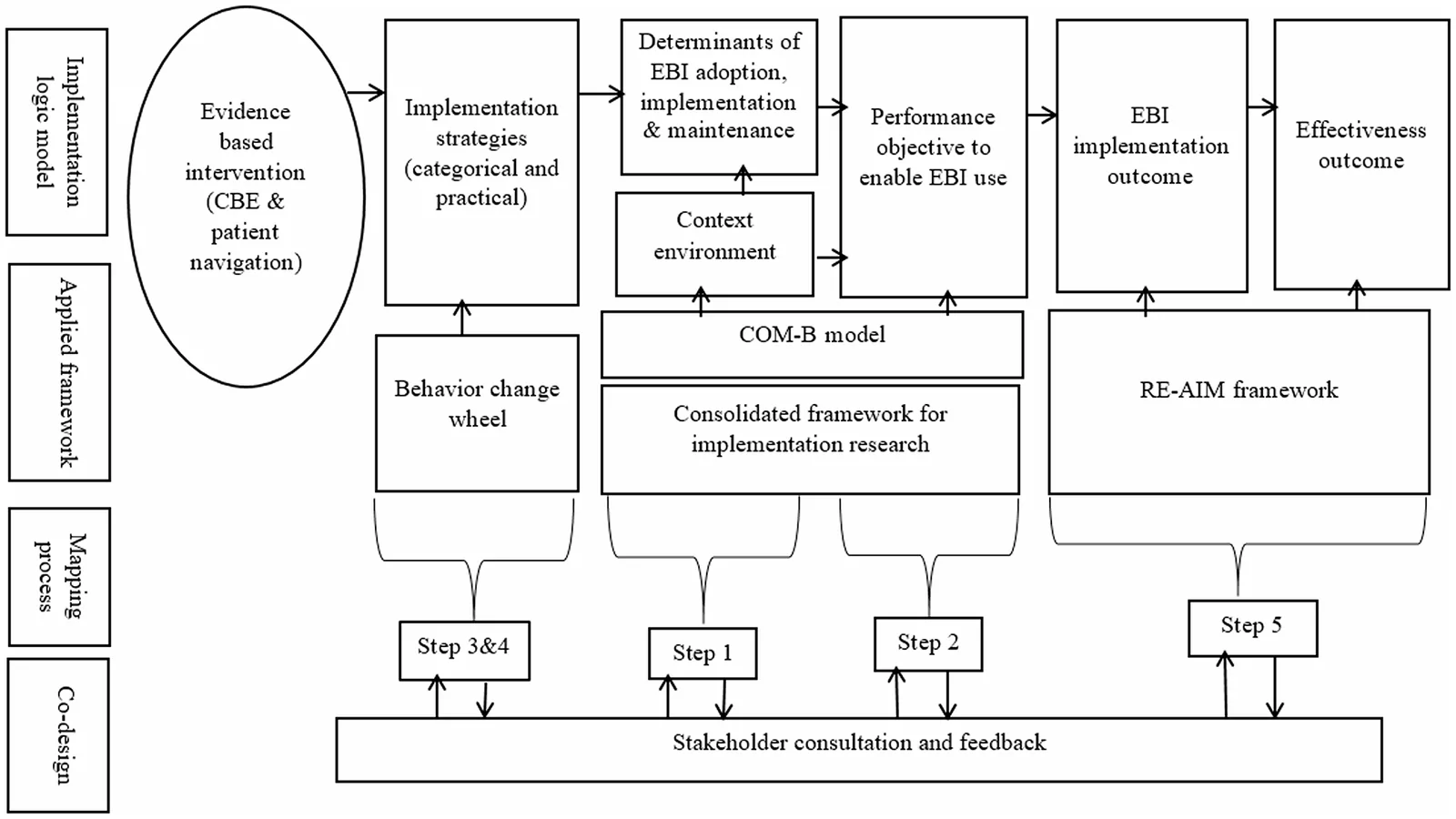
Clinical breast examination and patient navigation logic model for feasibility pilot trial implementation in sub Saharan Africa, adopted from Fernandez ME, et al., 2019

The study will employ a pragmatic, mixed-methods, single-arm pilot design that will be conducted in the four sub-Saharan African countries representing the NORA consortium: Ethiopia, South Africa, Nigeria, and Tanzania. The study follows a hybrid type 3 implementation-effectiveness approach, prioritizing implementation outcomes while assessing preliminary feasibility, acceptability and effectiveness indicators. The protocol is organized according to the five steps of the IM process: (1) conduct a needs and assets assessment and identify program adopters, implementers, maintainers, and champions; (2) identify adoption, implementation, and maintenance outcomes, along with performance objectives and determinants, and create matrices of change objectives; (3) select theoretical and practical implementation strategies to support those change objectives; (4) produce implementation protocols and materials; and (5) evaluate implementation feasibility, acceptability and preliminary effectiveness outcomes (Fig. 1).

### Study setting

The study will be conducted in primary health care or secondary care facilities across the four SSA countries of the Network for Oncology Research in Africa (NORA) consortium: Ethiopia, South Africa, Nigeria, and Tanzania. These countries represent diverse health system contexts seen across SSA. The participating facilities typically provide general outpatient care, maternal and child health services, and basic preventive services but have limited access to structured breast cancer screening programs and specialist oncology services. Facilities will be purposively selected based on criteria including patient volume, availability of referral pathways for diagnostic evaluation of breast abnormalities, and willingness to participate in the study. The implementation of CBE screening and PN will occur within each facility’s routine clinical workflows, allowing evaluation of the feasibility and implementation of these interventions within these real-world health system structures.

### Study population

Women will be eligible for screening if they (1) are within the age range recommended for breast cancer screening according to national guidelines in the participating countries, or as defined by the study team to reflect local epidemiological characteristics of the study populations, (2) are presenting to the participating facility for any health service unrelated to an active breast complaint (asymptomatic), and (3) are willing to undergo CBE following informed consent. Participants will be recruited directly in the study facilities with community and clinic-based awareness campaigns anticipated as part of our eventual implementation strategy set. Women presenting to study facilities with a known breast symptom will not be screened in the strict sense, but will instead be evaluated by HCPs trained in CBE, treated or referred according to local practice standards, and provided access to the PN services developed in this protocol. Data on both asymptomatic and symptomatic women will be captured and reported. Given differences in national cancer policy and health system organization, there will be some divergence between partner countries in screening setting, screening eligibility criteria, and anticipated sample size (Table 1).

**Table 1:** Areas of divergence by country in study settings, screening site and screening eligibility criteria, sub-Saharan Africa, 2026

Study country	Screening setting	Screening age eligibility	Estimated number of eligible women presenting to study facilities monthly
Ethiopia	General hospitals (4 sites)	Women age ≥ 30 years	~ 1415
South Africa	Primary health centers (4 sites)	Women aged 40–65 years	~ 1200
Nigeria	Primary health centers (2 sites)	Women aged 40–65 years	~ 500
Tanzania	Primary health centers (4 sites)	Women aged 40–65 years	~ 600

### Evidence-based intervention

This study will implement two evidence-based interventions to promote early detection and diagnosis of breast cancer in resource-constrained health settings: screening CBE and PN of women with abnormal screening exams to diagnostic and treatment services. Screening CBE will be provider-initiated and opportunistic, with exams being offered to all eligible women presenting to a study facility for any reason. PN for women needing follow-up testing will support successful referral patient education, referral coordination, and streamlined communication pathways. PN services will be provided by specially trained HCPs, likely nurses or lay health workers. Eligibility and frequency of CBE screening will conform to the national breast cancer guidelines for each country [24–27].

### Aim 1 (IM Steps 1–4): Implementation strategy development using implementation mapping

#### Step 1: Conduct a needs and assets assessment and identify program adopters, implementers, and recipients

We will conduct a separate needs analysis within each partner country’s study facilities, focused on defining existing resources and eliciting stakeholders’ perceived barriers and facilitators to implementation of screening CBE and PN. Representatives of each facility will first complete a resource survey. We will then conduct a series of semi-structured interviews with adopter and implementer type stakeholders (e.g., policymakers, facility administrators, physicians, nurses, and lay-health workers) and focus group discussions with recipient stakeholders (i.e., community members eligible for screening). The resource survey and qualitative discussion guides will be prepared with reference to the Consolidated Framework for Implementation Research (CFIR) to ensure attention to potential community-, clinic-, and individual-level determinants [29]. If additional actors responsible for adopting or implementing our interventions in the study facilities are identified through the course of data collection, we will aim to interview representatives from those groups as well. Use of the CFIR framework will ensure common determinant domains are assessed across all partner countries, but each country’s investigators will draft their own specific resource survey and qualitative discussion guides. Sample size for interviews and focus groups will be determined by the point of data saturation, again determined independently by each country’s investigators. Qualitative data will be analysed individually by each country’s study team and integrated with quantitative survey data to define perceived determinants of successful CBE and PN implementation.

#### Steps 2 and 3: Define participant-level adoption and implementation outcomes, performance objectives, key determinants, and change objectives. Select and design implementation strategies

Following the needs analysis, we will systematically define the factors that will influence successful implementation and the necessary changes at the study facilities. This information will be organized through a series of matrices prepared for each participant type involved in the intervention. For the first set of matrices, we will articulate adoption and implementation outcomes (i.e., straightforward implementation goals) and more granular performance objectives based on the key components of both evidence-based interventions. The next collection of matrices will link the performance objectives to the most impactful barriers and facilitators (i.e., determinants) of each. The determinants matrices will be organized according to the domains of the COM-B framework, which conceptualizes behavior as influenced by three domains: capability (the physical and psychological knowledge and skills necessary to perform a behavior), motivation (the mental processes that direct behavior), and opportunity (the environmental influences that affect behavior) [30]. We selected COM-B to organize the determinants for each participant because it describes determinants of behavior change at the individual level and the COM-B domains are linked to implementation strategy types through the Behavior Change Wheel, facilitating the final selection of implementation strategies.

In a final set of matrices, the identified determinants will be used to articulate corresponding change objectives (i.e., the individual, environmental, and policy changes required to meet our performance objectives). We will then use the behavior change wheel framework to select a set of implementation strategies supporting each change objective and corresponding to the relevant COM-B determinants. Theory-driven strategy types will be translated into practical strategies to align with local context, existing polices and available financial, human, and infrastructure resources. Though our strategy selection will ultimately be based on the findings from IM steps 1 and 2, we anticipate that strategies will be needed to support community engagement and breast cancer awareness, provider training on CBE and navigation skills, and ongoing motivational support strategies for adopting such services, designated space and time for screening, and routine monitoring with feedback mechanisms. Table 2 provides an abbreviated, hypothetical illustration of the three matrices series for clinic nurses.

**Table 2:** Abbreviated example of potential Implementation Mapping matrices series for implementers responsible for performing screening CBE in sub-Saharan Africa, 2026

Screening Nurse Matrix 1: Implementation Outcomes and Performance Objectives						
Target: Role	Implementation Outcomes				Performance Objectives	
Screening Nurse	The CBE screening nurse screens all eligible women for breast cancer according to study pilot protocol and follows clinical algorithm based on findings.				1. Performs a high-quality screening CBE for eligible women presenting to the screening clinic 2. Provides all women seen with basic breast health education 3. Ensures documentation for all women seen 4. Ensures referral documentation & instructions provided to all women referred for further evaluation of breast findings	
**Screening Nurse Matrix 2: Determinants and Change Objectives**						
	Determinants / Change Objectives					
Performance Objective	Capacity		Opportunity			Motivation
Performs a high-quality screening CBE for eligible women presenting to the screening clinic	1. Demonstrates ability to conduct a complete, high-quality CBE 2. Understands difference between assessment of asymptomatic women and women with breast symptoms 3. Able to practice and improve CBE skills over long term		1. Able to devote sufficient time to screening visit without neglecting waiting patients and other clinical and admin duties 2. Has a private examination space 3. Has sufficient space for patients waiting for screening			1. Understands rationale and potential health benefits of breast cancer screening 2. Expresses and maintains enthusiasm for participating in CBE screening program 3. Receives feedback on outcomes of women referred to tertiary after screening in CBE program 4. Feels recognized and appreciated for her contributions to the program
**Screening Nurse Matrix 3: Theoretical Change Methods and Practical Applications**						
Determinants / Change Objective		Behavior Change Wheel Intervention Type		Practical Applications		
1. Understands difference between assessment of asymptomatic women and women with breast symptoms 2. Understands rationale and potential health benefits of breast cancer screening		Education		1. Screening nurse will attend multi-day CBE training course, which includes didactic lectures on breast cancer burden, breast health basics, benefits of screening, and CBE 2. Will receive printed educational materials reflecting the above 3. Testing on breast health material at the end of didactic lectures with review of areas of weakness		
3. Demonstrates ability to conduct a complete, high-quality CBE		Training		4. Screening nurse will attend multi-day CBE training course including skills training in CBE with practice on mannequins and volunteer patients 5. Multi-day CBE training will also include standardized patient experiences including common screening scenarios and scenarios with patients with a clinical complaint 6. Training will also include experience with documentation, referral process, and data entry 7. Following demonstration of mastery via written tests and practical skills observation, research team will provide a certificate of training		
4. Able to practice and improve CBE skills over long term		Enablement		8. Department of Health and facility administrator commitment to maintain Screening Nurse in her position for full pilot period without rotation		

#### Step 4: Produce implementation protocols and materials

In this step, we will develop a set of research tools, including CBE and PN standard operating procedures, data collection tools, consent forms, and monitoring and evaluation tools. We will also prepare the materials needed to actually implement the intervention, such as health HCP and navigator education and training materials, CBE documentation forms, and referral tracking forms. All participant materials will be developed in English and the local languages to ensure accessibility and understanding.

### Aim 2 (IM Step 5): Pilot, refine, and evaluate implementation strategies

**Fig. 2:**
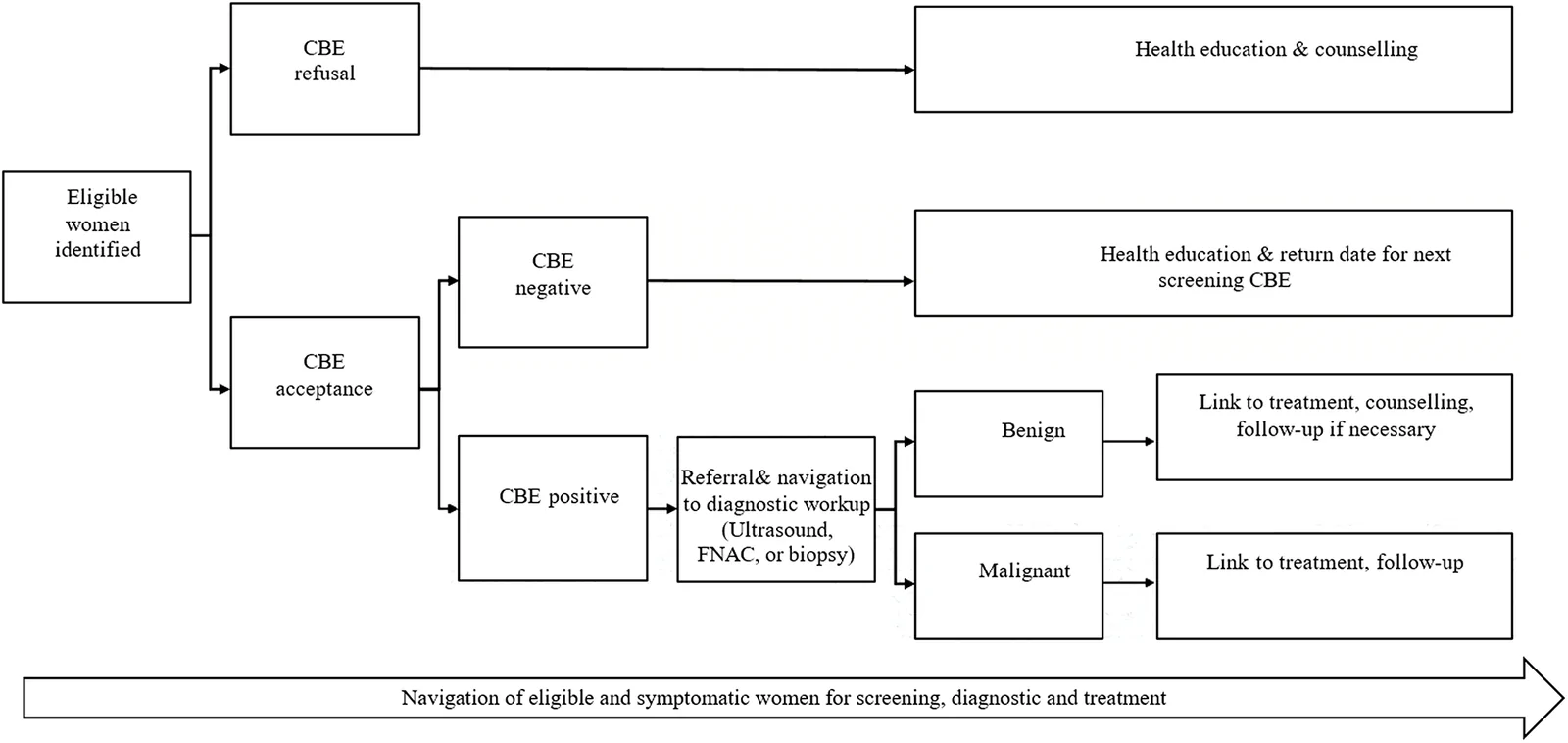
Flow chart showing the clinical breast examination (CBE) and patient navigation process for feasibility pilot trial implementation in sub Saharan Africa (adopted and modified from the Global Breast Cancer Initiative (GBCI)

**Fig. 3:**
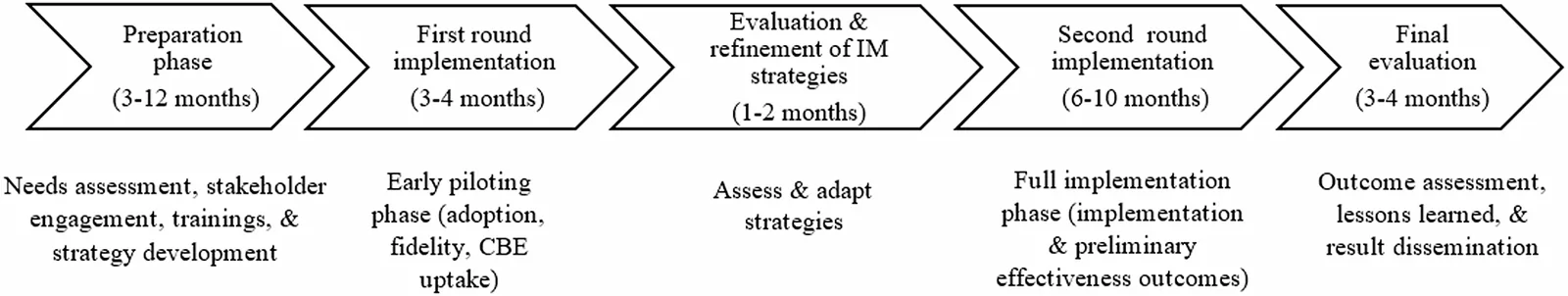
Evaluation and adaption time frame to implement provider-initiated CBE and patient navigation intervention in sub-Saharan Africa, 2026

In the final step in the implementation mapping process, we will iteratively pilot, refine, and evaluate the implementation strategies. Two sequential rounds of single-arm piloting and evaluation will be conducted at each of the health care facilities involved in implementation strategy development. Prior to the first round, participating HCPs at each of the facilities will receive training in CBE, PN, and the implementation process and will be deployed to provide CBE and navigation services based on the implementation strategies developed during Aim 1. A basic screening and navigation workflow is depicted in Fig. 2. Two rounds of evaluation will be conducted during piloting: a rapid evaluation after three to four months of piloting and a final evaluation at the end of the implementation period (Fig. 3). Evaluations at each time point will include quantitative and qualitative assessments and outcome measures will be guided by the RE-AIM framework [31]. Evaluation will include both process and outcome indicators to capture the implementation and preliminary effectiveness of the interventions within the facilities.

#### Quantitative data sources and collection

We will collect quantitative patient-level clinical and implementation process data from several sources to support our planned evaluations. For all eligible women who are willing to undergo CBE, the HCPs will directly capture basic socio-demographic characteristics, CBE findings, and clinical management recommendations. Navigators will track the navigation process using custom built research databases, capturing information on referral completion timing, diagnostic follow-up testing, recommended treatments with timing, and, when available, reasons for non-compliance or treatment abandonment among those lost to follow-up. Our CBE study records will only contain data on women who undergo screening, but all study facilities maintain central patient visit logs organized by date and containing the demographic data needed to identify all screening eligible women presenting to the facilities during the implementation periods. We will also collect data purely for implementation evaluation, including patient surveys and direct observation of implementing clinic staff, as described below.

All patient-facing data collection tools will be translated into local languages. HCPs and research staff involved with data collection will undergo training, and we will perform routine checks for completeness. Redcap data collection software, which allows for real-time data entry, secure storage, and ongoing monitoring of progress, will be used to collect data during screening CBE patient encounters and during the navigation process [41]. Periodic data quality audits will be carried out by the central research team to cross-verify selected records against source documents. Regular meetings with navigators and screeners, monitoring visits, and stakeholder advisory board meetings will be held to assess study progress and arising challenges.

#### Interim rapid evaluation

After the first three to four months of implementation, we will conduct a rapid evaluation focused on outcomes related to reach, adoption, and implementation with the goal of identifying early barriers to implementation and identifying areas requiring new or modified strategies (Table 3).

**Table 3:** Implementation and effectiveness evaluation outcomes description and data source per RE-AIM framework

Outcome Domains and Descriptions	Data Source
**Reach**	
Proportion of eligible women offered CBE	Patient exit surveys
Proportion of eligible women who undergo CBE	Random sampling of eligible women from facility visit registry with medical record and internal study records
Number of women referred for tertiary evaluation who received navigation service	Internal study records
Number of women screened per day	Internal study records
**Effectiveness**	
Number and proportion of screened women with an abnormal breast examination	Internal study records
Proportion of referred women contacted by navigator ever and proportion contacted within 2 days of referral	Internal study records
Proportion of referred women presenting to referral point at least once	Referral point medical records and internal study records
Median days from referral to presentation at referral point	Referral point medical records and internal study records
Proportion of women diagnosed with breast cancer completing diagnostic evaluation within 60 days of screening (diagnostic interval)	Referral point medical records and internal study records
Proportion of women diagnosed with a new benign breast lesion	Referral point medical records and internal study records
Proportion of women diagnosed with a new breast cancer	Referral point medical records and internal study records
Stage distribution of newly diagnosed breast cancers	Referral point medical records and internal study records
Proportion of women initiating breast cancer treatment within 30 days of confirmed diagnosis (treatment interval)	Referral point medical records and internal study records
**Adoption**	
Proportion of clinical staff at the end of the implementation period trained in intervention procedures/workflow	Study training logs and facility staff log
Proportion of clinical staff who adopt the intervention (offer and refer eligible women to screening)	Staff surveys and internal study records regarding screening referral source
**Implementation**	
Proportion of observed CBE exams where ≥ 80% of required components are performed	Direct observation of the trained nurse (screeners) while performing CBE using a structured observation checklist
Proportion of patients referred for tertiary evaluation who attend a tertiary appointment	Internal study records
Time spent on screening encounters per woman	Direct observation of the trained nurse (screeners) while performing a screening visit

Our primary implementation outcome is related to the intervention’s reach: the proportion of eligible women presenting to each study facility who undergo screening CBE. To estimate reach, we will use each facility’s central patient-visit registry database to randomly select 50 CBE-eligible women who visited the outpatient department for other medical care during the evaluation period. Women presenting to the facility specifically for evaluation of a breast complaint will be excluded. Our study database will then be cross-referenced to determine which of these women underwent a screening CBE. Reach will also be assessed through measuring the proportion of eligible women offered or referred to screening CBE regardless of their actual decision to accept screening. Research staff will gather this data through patient exit interviews on random days during the implementation period.

We will evaluate adoption of the intervention by clinical staff by measuring the proportion of current staff at the end of the first implementation period who underwent initial training. We will also examine daily clinical records to measure the proportion of eligible women referred for screening by individual HCPs. To assess quality of implementation, HCPs who perform screening CBE and navigation services will be directly observed using a CBE procedure checklist to capture performance to essential components of examination and counselling. The duration of each examination will also be recorded.

Each of the quantitative outcome measures will be compared with benchmark targets stated for individual countries (Table 4), and areas falling below target will be further explored through qualitative interviews with program adopters, implementers, and recipients, drawing from the same types of participants included in the Step 1 needs assessment. Participants will be asked about their perceived perceptions on feasibility and acceptability and associated enablers and barriers to performing specific aspects of the CBE and PN intervention, and we will again use the COM-B framework to organize discussion guides for these interviews.

**Table 4:** Target benchmarks of each individual country for quantitative implementation measures for refinement of evaluation based on RE-AIM framework

Measure	Benchmark			
**Reach**	South Africa	Ethiopia	Nigeria	Tanzania
Proportion of eligible patients offered CBE	≥ 70%	≥ 50%	≥ 50%	≥ 50%
Proportion of eligible patients who undergo CBE	≥ 50%	≥ 25%	≥ 25%	≥ 25%
**Adoption (Staff Level)**				
Proportion of clinical staff oriented to implementation procedures/workflow	≥ 75%	≥ 50%	≥ 50%	≥ 50%
Proportion of clinical staffs adopt the intervention (offer and refer eligible women to screening)	≥ 75%	≥ 50%	≥ 50%	≥ 50%
**Implementation**				
Proportion of observed CBE exams where ≥ 80% of required components are performed	≥ 80%	≥ 80%	≥ 80%	≥ 80%
Proportion of patients referred for tertiary evaluation who attend a tertiary appointment	≥ 80%	≥ 80%	≥ 80%	≥ 80%
Median time spent on screening encounter per woman	≤ 5 min	≤ 5 min	≤ 5 min	≤ 5 min

Findings from this interim evaluation will be systematically reviewed, including using rapid qualitative research methods to identify strengths, challenges, and areas requiring improvement. Based on this evaluation, our suite of implementation strategies and workflow processes will be adjusted to enhance their reach, adoption and implementation. The refined strategies will then be formally communicated to the study facilities and staff will undergo short-form training in any changes.

#### Final evaluation

Following strategy refinement, we will pilot implementation for a further six to twelve months, followed by a final evaluation. All the measures and data collection approaches used above for rapid evaluation will be repeated during this final evaluation. To compare the proportion of eligible women undergoing screening CBE during the final evaluation period versus prior to implementation, we will collect data on screening rates in a sample of women who visited each facility during the six months prior to the start of our intervention. To do this, we will use the facilities’ central patient visit registries to identify 50 screening eligible women from the pre-intervention period and then review their individual medical records for any documentation of a breast examination on the day of their visit or within one year prior. Again, women presenting to the facility seeking evaluation of a specific breast complaint will be excluded from these samples. In addition, we will collect preliminary data on the clinical effectiveness of CBE and PN, measuring time to diagnosis, stage at diagnosis, and time to initiation treatment among women diagnosed with breast cancer through the program (Table 3). Data will be collected from patients’ primary medical records. Qualitative interviews and focus groups with adopters, implementers, and recipients will focus on general experience with the CBE and PN intervention, ongoing barriers to reaching all eligible women for screening, full adoption and complete implementation of the intervention by facility staff members, and anticipated challenges with maintaining the program over time.

### Data analysis

After data cleaning, the proportion of sampled eligible women undergoing screening CBE in the six months before implementation and during the final evaluation period will be compared using Fisher’s exact test. Other quantitative implementation outcomes will be summarize using descriptive statistics such as the mean, median, standard deviation, and range. Qualitative data from interview and focus group will be analyzed using a combined inductive/deductive approach. Codes will be identified directly from the transcript data inductively and themes within those codes will be organized deductively according to the domains and constructs of the CFIR [29] and COM-B/BCW [30] frameworks. We will adhere to the Standards for Reporting Qualitative Research (SRQR) to ensure transparency and accuracy throughout qualitative data collection, analyses, and reporting [32].

### Stakeholder advisory boards

Each of the study countries will have their own stakeholder advisory board (SAB), consisting of representatives from national or regional health governing bodies, study facility administrators, local oncology specialist providers, primary HCPs from study facilities, breast cancer survivors, and local NGO partners. Each country’s SAB will be recruited at the outset of the study and will be consulted throughout the study through regular meetings with the investigators. The SAB will advise at each step of the IM process, including review of needs assessment data collection tools, validity of needs assessment findings, initial selection and refinement of implementation strategies, appropriateness of implementation materials, and interpretation of findings from the piloting evaluation. The SAB will also help the study team liaise with staff at the study facility, the wider community, and policymakers for introduction of the study and dissemination of study findings.

## Discussion

Our implementation feasibility pilot trial protocol outlines a structured, theory driven approach to implementing a pair of evidence-based interventions aimed at improving early detection of breast cancer and ensuring timely diagnosis and treatment initiation through in SSA: provider-initiated CBE and PN. We will apply the IM process [28] to intentionally develop, adapt, and evaluate implementation strategies that are practical, scalable, and sustainable in the low and middle-income setting, where structured breast cancer screening and navigation services are very limited [33]. Using the IM process specifically allows us to integrate stakeholder engagement, contextual assessment, and evidence-based strategy development to increase the likelihood that our approach to implementation is feasible, acceptable, and sustainable within the available resources.

Provider-initiated CBE is an evidence-based approach to integrating breast screening into routine outpatient care, rather than relying solely on patient-initiated complaints. This approach could be especially vital in these settings with low health literacy, misconceptions around cancer, and limited awareness of breast health [34–36]. The participation of nurses to facilitate cancer care is recommended in developing settings due to the shortage of professionals trained in cancer diagnosis and treatment. In Rwanda, CBE done by trained nurses at primary care clinics improved the detection of early-stage disease [37]. Similarly, a randomised controlled trial in Ethiopia found that the involvement of trained nurses improved the adherence of patients with breast cancer to endocrine treatment [38]. This implies the importance of utilising the nurses or other trained staff to enhance early detection practices in resource-limited settings facing a shortage of professionals who specialise in cancer.

Findings from a situational assessment conducted the Ethiopian researchers in our consortium provide an important empirical foundation for this study, indicating that CBE and PN are feasible and acceptable when delivered using existing human resources and infrastructure [39]. These findings suggest that task-sharing approaches, integration within primary health care services, and leveraging existing referral pathways can support effective implementation without requiring substantial additional resources. Our assessment also highlighted critical enabling factors, including community engagement, collaboration, and integration of screening and navigation services into routine clinical workflows. The needs assessment in Ethiopia and other partner countries will build on these insights. We anticipate that contextually tailored implementation strategies addressing these factors will be essential to achieving consistent delivery and minimizing variability across implementation sites.

Our initial evaluation after three to four months of implementation piloting is intentionally designed for refinement of strategies based on early stakeholder experience and persistent barriers. This approach aligns with implementation science best practices, recognizing that early identification of implementation challenges, such as workflow misalignment, training gaps, or incomplete communication, can prevent compounding inefficiencies late in the implementation period. Following these early efforts to refine and stabilize our strategies, the final evaluation will provide a comprehensive assessment of implementation success and a preliminary impression of screening CBE’s and navigation’s ability to aid early detection and timely diagnosis of breast cancer across a group of similar novel settings. Together, these two rounds of evaluation offer a pragmatic framework for iterative improvement. Conducting this feasibility study across four sub-Saharan African countries within the NORA consortium also enhances both the external validity and scalability of the findings. While health system contexts vary across consortium countries, many face shared challenges, including limited diagnostic capacity, delayed presentation, and fragmented referral systems [5, 35, 40, 41]. By documenting context-specific adaptations and implementation strategies across diverse settings, this pilot trial will generate transferable knowledge that may inform scale-up in other low and middle-income settings facing similar constraints. Moreover, our preliminary efficacy findings are expected to inform a future, larger hybrid effectiveness implementation trial. Ultimately, our aims and protocol design align with the mandate of WHO’s Global Breast Cancer Initiative and our partner countries’ national cancer control guidelines’ recommendations for improving the early detection and diagnosis of breast cancer.

## Conclusion

This multicounty feasibility pilot study will develop, pilot, and evaluate implementation strategies in various health care settings in four sub-Saharan African countries that can be incorporated into routine health care practice to address multiple points of delay in the breast cancer care pathway, mainly early detection, diagnosis, and referral. The findings will offer promising strategies to address critical delays in the breast cancer care continuum for low- and middle-income settings that face similar challenges.

## Supplementary Information

Below is the link to the electronic supplementary material.

Supplementary Material 1

## Data Availability

No datasets were generated or analysed during the current study.

## Abbreviations

CBE: Clinical breast examination
CFIR: Consolidated framework for implementation research
GBCI: Global breast health initiative
HCP: Health care provider
HEW: Health extension worker
IDI: In-depth interview
IM: Implementation mapping
LMIC: low- and middle-income countries
PN: Patient navigation
RE-AIM: Reach, effectiveness, adoption, implementation, and maintenance framework
SPH: School of public health
VIF: Variance inflation factor
WHO: World Health Organization
